# Ontogenetic variations of proteins in neurons of the lateral preoptic nucleus of rats' hypothalamus under a modified light regime

**DOI:** 10.25122/jml-2023-0049

**Published:** 2023-04

**Authors:** Roman Yevgenovych Bulyk, Vladyslav Romanovych Yosypenko, Tetiana Vasylivna Protsak, Mariana Ivanivna Kryvchanska, Kateryna Vasylivna Vlasova, Volodymyr Leonidovych Voloshyn, Oleksiy Vasyliovych Smetanyuk, Yuliana Ruslanivna Lukan, Michael Ivanovych Sheremet, Tetyana Sergiivna Bulyk, Dmytro Volodymyrovych Proniaiev, Larysa Vasylivna Rynzhuk, Maryna Dmitrivna Gresko, Mykhailo Mykhailovych Gresko, Yuriy Vasyliovych Tovkach, Olha-Anna Romanivna Savytska

**Affiliations:** 1 Department of Medical Biology and Genetics, Bukovinian State Medical University, Chernivtsi, Ukraine; 2 Department of Human Anatomy named by M. H. Turkevych, Bukovinian State Medical University, Chernivtsi, Ukraine; 3 Department of Medical and Pharmaceutical Chemistry, Bukovinian State Medical University, Chernivtsi, Ukraine; 4 Department of Surgery No.1, Bukovinian State Medical University, Chernivtsi, Ukraine; 5 Department of Obstetrics and Gynecology, Bukovinian State Medical University, Chernivtsi, Ukraine; 6 Department of Anatomy, Clinical Anatomy, and Operative Surgery, Bukovinian State Medical University, Chernivtsi, Ukraine; 7 Faculty of Medicine, Bukovinian State Medical University, Chernivtsi, Ukraine

**Keywords:** circadian rhythms, hypothalamus, lateral preoptic nucleus, proteins, melatonin, photoperiod

## Abstract

Proteins can be key biochemical markers for evaluating the functional activity of nervous system cells. They are involved in the proliferation and differentiation of nerve and glial cells and the arrangement of many metabolic functions of the brain. This study aimed to analyze the concentration of proteins in the neurons of the lateral preoptic nucleus (LPON) of the hypothalamus in mature and old rats under standard and altered lighting conditions. Our results show that the concentration of proteins in mature rats was significantly higher than in old rats (0.274±0.0017 optical density units), with a predominance of carboxyl groups, indicating a high intensity of protein metabolism. Additionally, we found that changes in the lighting regime have a differential effect on the optical density of specific staining for protein in LPON neurons. Specifically, light deprivation did not significantly alter the optical density of specific staining for protein in neurons of the hypothalamus LPON of mature rats regardless of the period of the day, while in old rats, the intensity of protein staining decreased. Light exposure, on the other hand, led to an increase in the average color intensity for protein in neurons of the hypothalamus LPON in mature rats (0.326±0.0014 optical density units), while in old rats, a decrease in the average color intensity for protein in neurons of the hypothalamus LPON was observed (0.196±0.0017 optical density units).

## INTRODUCTION

Biological rhythms are cyclic fluctuations that occur in almost all physiological processes in living systems [1,2]. Any changes in the environment or lifestyle can be a source of stress, which is a universal response to the need to adapt to new conditions [3]. Synchronizers are factors that affect the rhythm of these processes, and light and darkness are among the strongest synchronizers for humans [4]. The daily exposure of the retina to light is essential for synchronizing circadian rhythms with the external 24-hour solar environment [5]. Circadian rhythms become less stable with aging, characterized by a decrease in amplitude and the ability to adapt to a phase shift (changes in the phases of circadian rhythms) [6].

The sleep-wake cycle is the most obvious circadian rhythm observed in humans and many animals [7,8]. Regular sleep is one of the three pillars of health, next to proper nutrition and physical activity [9]. On average, humans spend approximately one-third of their lives sleeping, and the quality of sleep determines the overall level of health [10]. The transition between wakefulness and sleep and vice versa is regulated by activating or inhibiting certain neurotransmitter systems. For instance, the monoaminergic systems of the brainstem, the cholinergic system of the anterior parts of the brain [11], and the orexinergic system [12] are involved in maintaining the state of wakefulness. At the same time, the tuberomammillary nucleus of the hypothalamus plays the leading role in maintaining wakefulness [13]. The lateral preoptic nucleus (LPON) of the hypothalamus, a structure within the anterior hypothalamus, is a critical component in regulating the sleep-wake cycle [14,15]. The LPON neurons contain neurotransmitters such as galanin and gamma-aminobutyric acid [16], which provide inhibitory innervation of the main monoamine systems that maintain wakefulness [17].

Sleep is fundamental for the mental and physical health of humans [18]. Disruptions in circadian rhythms can lead to inconsistencies between the sleep period and the physical/social 24-hour environment cycle. This inconsistency can result in sleep disorders such as insomnia at night and sleepiness during the day [7]. A decrease in the duration and/or quality of sleep gradually leads to changes in nervous and neuroendocrine functions, including an increased level of stress, cognitive and metabolic disorders, and increased risk of developing cancer, metabolic, cardiovascular diseases, and premature death [19]. The global population of elderly individuals is increasing, and aging is associated with numerous physiological changes, including changes in sleep patterns [20,21].

Age, psychological and physiological conditions, culture, and environmental factors influence sleep time and quality [22]. Among these factors, lighting conditions are particularly important. Changes in lighting intensity, such as a decrease in the morning and an increase in the evening [7], as well as a nighttime human activity, are common causes of disruptions in the sleep-wake rhythm [23]. These changes are accompanied by the inhibition of melatonin synthesis and secretion [24], which can contribute to the development of desynchronosis [25].

Therefore, the objective of the study was to investigate the concentration of proteins in neurons of the hypothalamus LPON in mature and old rats under standard and altered lighting conditions.

## Material and methods

The experiments were conducted on white non-linear mature and old rats. The experimental animals were divided into six groups, each consisting of two subgroups of six animals. Group 1 consisted of mature rats kept under standard light conditions for seven days (lighting with fluorescent lamps from 8 am through 8 pm, illumination level – 500 lux). Group 2 consisted of mature rats kept in round-the-clock darkness (light deprivation) for the same period. Group 3 consisted of mature rats kept for seven days in conditions of round-the-clock illumination (light stimulation). Group 4 consisted of old rats kept under a standard light regime (the level and time of illumination were the same as in mature rats) for seven days. Group 5 consisted of old rats kept under round-the-clock darkness for seven days. Finally, group 6 consisted of old rats kept under conditions of round-the-clock lighting for the same period.

Tissue samples were collected at 12-hour intervals (at 2 pm and 2 am) by decapitation under etaminal anesthesia (40.0 mg/kg, intraperitoneally) to identify circadian differences. All stages of the experiment were carried out in compliance with the orders of the Ministry of Health No. 690 of September 23, 2009, No. 944 of December 14, 2009, No. 616 of August 3, 2012, and the laws of Ukraine, EEC Directive No. 609 (November 24, 1986), Convention of the Council of Europe on the Protection of Vertebrate Animals Used in Experiments and Other Research Purposes (March 18, 1986) and the main provisions of the Decision of the First National Congress on Bioethics “General Ethical Principles of Animal Experiments” (2001).

The brain was fixed in neutral buffered 10% formalin solution for 22-24 hours after removal, before cutting the material, with small incisions made in the pia mater for uniform and faster soaking. This preliminary fixation allowed the brain tissue to be cut without deformations and enabled histochemical studies, ensuring that the proteins retained their chemical and antigenic properties. After fixation, the whole brain was sliced into approximately one-millimeter-thick sections. The first slice passed through the anterior part of the optic chiasm, and the second slice passed through the posterior part. These slices were then dehydrated in an ascending battery of alcohols, embedded in paraffin at 58 ºC, and cut into serial 5 µm-thick sections using a sledge microtome.

Histochemical studies of rat hypothalamus LPON proteins were carried out using histochemical staining with bromophenol blue for proteins according to the Mikel Calvo method. Digital copies of images of histochemical preparations were obtained using a Delta Optical Evolution 100 microscope with plan achromat objectives in accordance with the required optical magnification and an Olympus SP550UZ digital camera. These images were stained according to Mikel Calvo and were digitally converted into monochromatic images with 256 gradations of their shades. The intensity of staining (optical density) for protein was studied using the method of computer micro densitometry on monochromatic images, which allowed for the protein concentration in certain locations of neurons to be determined.

The resulting digital data were statistically processed using the PAST computer program for statistical calculations [26]. A preliminary check for the normality of distribution was applied using the Wilkie-Khan-Shapiro test. The hypothesis of the distribution normality was not rejected in all statistical samples; therefore, the arithmetic mean, its error (M±m), and odd two-sided Student's t-test were calculated. Additionally, the Mann-Whitney test was used to improve the reliability of the results of checking the differences between the study groups in the average trends, considering the relatively small number in the statistical samples.

## Results

The Mikel Calvo technique produced clear and distinct staining of neurons in the hypothalamus LPON of all rats, irrespective of age. The staining had a polymorphic character, with the presence of small or large granules and diffuse background, reflecting the distribution of proteins in the organelles and cytosol of these cells.

The optical density of the specific color for protein in the monochromatic version in LPON neurons of the hypothalamus of mature and old rats under normal lighting conditions was measured using the Mikel Calvo histochemical method (Table 1).

**Table 1:** The optical density of specific protein staining in neurons of rats' hypothalamus LPON under standard light conditions.

Time	Optical density of histochemical staining for protein (in optical density units)	
	Mature rats	Old rats
**2 pm**	0.274±0.0017	0.222±0.0014 (p<0.001)
**2 am**	0.271±0.0016	0.221±0.0013 (p<0.001)

p – is the probability of difference compared to mature rats.

The concentration of proteins in the neurons of the hypothalamus LPON of mature rats was significantly higher than that of old rats, as indicated by the optical density of staining (p<0.001) (Table 1). Additionally, the staining intensity of neurons in different zones of the hypothalamus LPON, whether in the central or peripheral areas, was consistent among mature rats, with no significant variation in the distribution of granular and diffuse material.

In contrast, old rats showed a unique staining pattern, where some neurons displayed intense protein staining similar to that of most neurons in mature rats. These neurons were predominantly located in the central parts of the hypothalamus LPON, although they were not uniformly mixed with neurons exhibiting a weaker staining intensity.

These findings suggest that aging may affect the distribution and intensity of protein accumulation in the neurons of the hypothalamus LPON.

The optical density of specific protein staining in the monochromatic variant in neurons of the hypothalamus LPON of rats under light deprivation using the Mikel Calvo histochemical method is presented in Table 2.

**Table 2:** The optical density of specific protein staining in neurons of rats' hypothalamus LPON under light deprivation.

Time	Optical density of histochemical staining for protein (in optical density units)	
	Mature rats	Old rats
**2 pm**	0.273±0.0018	0.208±0.0016 (p<0.001)
**2 am**	0.276±0.0015	0.214±0.0015 (p<0.001)

p – is the probability of difference compared to mature rats.

The relative concentration of proteins in the neurons of the hypothalamus LPON of mature rats did not significantly change, regardless of the period of the day, while the intensity of protein staining in the hypothalamus LPON neurons significantly decreased in old rats. As mentioned above, the intensity of protein staining in the hypothalamus LPON neurons of old rats was markedly lower during light deprivation, particularly at 2 pm compared to 2 am.

It is worth noting that in mature rats, the staining of neurons in different areas of the nucleus was uniform at 2 am but not at 2 pm, where there was variability in color intensity among neurons. On the other hand, during light deprivation at 2 pm in old rats, the staining of neurons in the hypothalamus LPON was more uniform, but at 2 am, there was significant variability in the intensity of staining, regardless of whether the neurons were in the central or peripheral zones of the nucleus.

Table 3 presents the optical density of the specific protein staining for protein in the monochromatic version using the Mikel Calvo histochemical method in neurons of the hypothalamus LPON of mature and old rats under light stimulation.

**Table 3:** The optical density of specific protein staining in neurons of rats' hypothalamus LPON under light stimulation.

Time	Optical density of histochemical staining for protein (in optical density units)	
	Mature rats	Old rats
**2 pm**	0.321±0.0017	0.198±0.0016 (p<0.001)
**2 am**	0.326±0.0014	0.196±0.0017 (p<0.001)

p – is the probability of difference compared to mature rats.

The results suggest that the response of rats to changes in lighting conditions varies depending on their age. In mature rats, the average intensity of protein staining in the neurons of the hypothalamus LPON increased, while in old rats, a decrease in the average intensity of protein staining was observed. Furthermore, mature rats had uniformly stained neurons for protein in both the central and peripheral zones of the hypothalamus LPON during both periods of the day, whereas in old rats, uneven staining was observed at 2 pm but not at 2 am.

## Discussion

In this study, we demonstrated that the concentration of proteins in the neurons of the hypothalamus LPON of mature rats was significantly higher than that of old rats (p<0.001). There was no significant difference in protein concentration between the two age groups at different periods of the day (2 pm and 2 am). However, changes in the lighting regime had different effects on the processes of protein accumulation in the neurons of the rat hypothalamus LPON. Specifically, in mature rats, the concentration of proteins in the hypothalamus LPON neurons did not change on average during light deprivation, regardless of the time of day, whereas in old rats, the concentration of proteins significantly decreased (p<0.001). Although prolonged illumination is known to be a significant stressor and a trigger for desynchronosis [27], our findings suggest that the effect of light stimulation on the concentration of proteins in the neurons of the hypothalamus LPON depends on age.

Interestingly, mature and old rats responded in opposite ways to light stimulation regarding the relative concentration of proteins in the hypothalamus LPON neurons. Specifically, we observed a significant increase in protein concentration in mature rats, while a significant decrease was observed in old rats (p<0.001). The observed difference in the processes of protein accumulation between mature and old rats in the neurons of the hypothalamus LPON can be explained by the decrease in the body's compensatory capabilities in old rats and the decrease in melatonin concentration due to decrease in the total number of pinealocytes and the development of pineal gland sclerosis [28]. Additionally, the changes in the architectonics of LPON neurons against the background of changes in lighting regime and light stress may also contribute to the decrease in the concentration of proteins in the neurons of the hypothalamus LPON of old rats. Specifically, our previous studies have established that more apparent changes in the ultrastructures of LPON neurons were caused by light stimulation, namely, the thickening of the cytoplasm and destructive changes in the organelles of synthetic and energy supply against the background of the long light period [29].

## Conclusion

The optical density of specific staining for protein using the Mikel Calvo histochemical method in the monochromatic version was significantly higher in mature rats than in old rats. Additionally, different lighting regimes had varying effects on the optical density of specific staining for protein in the neurons of the hypothalamus LPON of rats. Notably, light deprivation did not significantly alter the optical density of specific staining for protein in neurons of the hypothalamus LPON of mature rats, while it decreased the intensity of protein staining in old rats. Conversely, light stimulation led to an increase in the average staining for protein in the neurons of the hypothalamus LPON in mature rats and a decrease in the average color intensity for protein in old rats.

## Personal thanks

The authors would like to acknowledge the administration of Bukovinian State Medical University and the chief of the Department of Pathological Anatomy, Professor Igor Davydenko.
